# Qiling decoction enhances the anti-tumor activity of abiraterone acetate by up-regulating miR-143 expression in abiraterone acetate-resistant prostate cancer cells

**DOI:** 10.3389/fmed.2025.1643506

**Published:** 2025-12-09

**Authors:** Lizhi Chen, Hongwen Cao, Peng Sun, Yigeng Feng, Lei Chen, Renjie Gao

**Affiliations:** Surgical Department I (Urology Department), LONGHUA Hospital Shanghai University of Traditional Chinese Medicine, Shanghai, China

**Keywords:** abiraterone acetate, autophagy, drug resistance, CRPC, QLD

## Abstract

**Introduction:**

Abiraterone acetate is a key therapeutic agent for castration-resistant prostate cancer (CRPC), but the cancer resistance limits its long-term efficacy. While several mechanisms of abiraterone acetate resistance have been proposed, the role of microRNAs (miRNAs) in this process remains incompletely understood. Here the aim of the study was to investigate miR-143 as a potential tumor suppressor in prostate cancer, and elucidate its involvement in abiraterone acetate resistance. Additionally, Qiling decoction (QLD), a traditional Chinese medicine formulation, was tested for its ability to restore miR-143 expression and enhance abiraterone efficacy.

**Methods:**

Abiraterone acetate-resistant prostate cancer (PC) cell lines, PC3-AbiR and DU145-AbiR, were established through long-term abiraterone exposure. The expression of miR-143 was analyzed using qRT-PCR, and its effects on the JNK/p-Bcl2-Beclin1 signaling axis were examined via Western blot and co-immunoprecipitation assays. Functional experiments, including CCK-8 assays, were carried out to evaluate how miR-143 modulation affects abiraterone acetate sensitivity.

**Results:**

miR-143 expression was significantly downregulated in abiraterone acetate-resistant PC cells. Downregulation of miR-143 was shown to be linked with increased phosphorylation of JNK and p-Bcl2, along with elevated expression of Beclin1, indicating activation of the JNK/p-Bcl2-Beclin1 signaling axis. Functional studies revealed that miR-143 inhibition promoted cell survival and autophagy, while its overexpression restored abiraterone acetate sensitivity. Treatment with QLD upregulated miR-143 expression, suppressed JNK/p-Bcl2-Beclin1 signaling, and enhanced abiraterone acetate-induced cytotoxicity. Inhibition of miR-143 abolished the effects of QLD, confirming its central role in mediating abiraterone acetate resistance. These findings demonstrate that miR-143 downregulation contributes to abiraterone acetate resistance in prostate cancer by activating the JNK/p-Bcl2-Beclin1 signaling axis and promoting autophagy.

**Conclusion:**

Restoration of miR-143 expression through QLD treatment enhances abiraterone acetate sensitivity, suggesting a potential therapeutic strategy for overcoming drug resistance in CRPC.

## Introduction

Prostate cancer is the second most common malignancy among men worldwide and remains a leading cause of cancer-related mortality (1). Androgen deprivation therapy (ADT) has been the cornerstone for treating advanced prostate cancer (2); however, nearly all patients eventually develop castration-resistant prostate cancer (CRPC), a highly aggressive form that continues to progress despite androgen suppression (3). The introduction of androgen receptor (AR)-targeting agents, such as abiraterone acetate, has significantly improved survival in patients with CRPC by inhibiting androgen biosynthesis from the adrenal glands, testes, and tumor cells. However, acquired resistance to abiraterone acetate occurs in most patients, ultimately leading to disease progression and poor prognosis. The underlying mechanisms of abiraterone acetate resistance remain incompletely understood, posing a significant challenge to the effective management of CRPC (4–6).

Previous studies have implicated multiple molecular pathways in the development of abiraterone acetate resistance. One key mechanism involves the restoration of AR signaling through alternative splicing, mutations, or intratumoral androgen synthesis (7–9). Other research has suggested that epithelial-mesenchymal transition (EMT) (10), metabolic reprogramming, and activation of survival pathways, such as the PI3K/AKT and MAPK signaling cascades (11), contribute to resistance. Recent findings have highlighted the significant roles of non-coding RNAs, particularly microRNAs (miRNAs), in regulating key oncogenic processes in prostate cancer (12). Among them, miR-143 has been identified as a tumor suppressor in various malignancies, including prostate cancer (13, 14). Several studies have shown that miR-143 expression is significantly reduced in CRPC and is associated with enhanced tumor growth, increased metastatic potential, and resistance to chemotherapy (13–15). However, its specific role in abiraterone acetate resistance and its downstream regulatory mechanisms remain poorly characterized.

One potential target of miR-143 is the JNK/p-Bcl2-Beclin1 signaling axis (16), which plays a critical role in cellular stress responses, apoptosis, and autophagy. Autophagy has been recognized as a key survival mechanism in prostate cancer cells, particularly under therapeutic pressure (16, 17). Studies have suggested that enhanced autophagy promotes resistance to AR-targeted therapies by protecting cancer cells from apoptosis (18). Dysregulation of the JNK/p-Bcl2-Beclin1 pathway has been implicated in autophagy-mediated chemoresistance in multiple cancer types, yet its role in abiraterone acetate resistance remains largely unexplored (16). Understanding the interplay between miR-143 and this pathway could provide novel insights into overcoming drug resistance.

Despite advances in targeted therapies, there remains a clinical gap in the effective management of abiraterone acetate-resistant prostate cancer. There is an urgent need for alternative strategies that can overcome resistance and improve therapeutic efficacy. Traditional Chinese medicine formulations (19), such as Qiling decoction (QLD), have gained attention for their potential in cancer treatment (20). QLD has been reported to enhance chemotherapy sensitivity and modulate key signaling pathways in various malignancies (21). Recent studies suggest that QLD exerts anti-cancer effects by regulating apoptosis and autophagy (22); however, its role in reversing abiraterone acetate resistance in prostate cancer has not been investigated.

In this study, we aimed to determine the role of miR-143 in abiraterone acetate-resistant prostate cancer and its regulatory effects on the JNK/p-Bcl2-Beclin1 signaling axis. Furthermore, we explored the potential of QLD to restore miR-143 expression and sensitize abiraterone acetate-resistant cells to treatment. By elucidating these mechanisms, our study provides new insights into the molecular basis of abiraterone acetate resistance and suggests a potential therapeutic strategy for improving treatment outcomes in CRPC patients.

## Materials and Methods

### Cells, cell culture, and chemicals

Human prostate cancer cell lines PC3 and DU145 were obtained from ATCC (Manassas, VA, USA), and they were cultured in RPMI-1640 medium (Gibco) containing 10% fetal bovine serum (FBS, Gibco) and 1% penicillin-streptomycin (Beyotime, Shanghai, China; Cat. No. C0222). The incubation conditions were maintained at 37 °C with 5% CO2 in a humidified chamber.

Abiraterone acetate (Abi; Cat. No. S2246; Solvent: ethanol) and the JNK agonist Anisomycin (ANI; Cat. No. S7409; Solvent: DMSO) were purchased from Selleck, Houston, Texas, USA. The formulation of Qiling decoction (QLD) was used as described in our previous work (20), and the formulation of QLD was also applied for a Chinese invention patent (CN201710664737.7) by the authors. Briefly, QLD is a traditional Chinese medicinal formula composed of multiple herbs, including 15 g of *Astragalus mongholicus* Bunge (root), 15 g of Herba *Leonuri* (Herba), 15 g of Radix *Codonopsis* (root), 15 g of Radix Rehmanniae (root), 30 g of *Rabdosia rubescens* Hara (root), 9 g of Rhizoma Curcumae Longae (root), 15 g of *Solanum septemlobum* Bunge (seed) and 9 g of Radix Glycyrrhizae Preparata (Herba). These components are decocted together in 500 ml sterile water, following standard pharmacopeia guidelines. The aqueous extract is then filtered and lyophilized to obtain a powdered form suitable for *in vitro* studies. In this study, QLD powder was dissolved in sterile water and filtered through a 0.22 μm membrane before application to cell cultures. The final working concentration was 10 μg/mL unless otherwise specified. All our experiments were carried out with the same batch.

### Development of abiraterone acetate-resistant cell lines

To induce abiraterone acetate resistance (PC3-AbiR and DU145-AbiR), parental cells were exposed to progressively increasing doses of abiraterone acetate, beginning at 1 μM and increasing to 20 μM over a 12-months period. Resistant sublines were subsequently maintained in 10 μM abiraterone acetate to preserve the phenotype.

To validate resistance, PC3, DU145, PC3-AbiR, and DU145-AbiR cells were incubated with medium containing escalating concentrations of abiraterone acetate (0 ∼ 20 μM) for 24 h. Cell viability was assessed via the CCK-8 assay, which confirmed enhanced survival of resistant cells compared to their parental counterparts.

### CCK-8 assay

Cell viability was determined using the Cell Counting Kit-8 (CCK-8) assay. PC3, DU145, PC3-AbiR, and DU145-AbiR cells were seeded in 96-well plates (5 × 10^3^ cells/well) and incubated overnight. Cells were then treated with different concentrations of abiraterone acetate (0 ∼ 20 μM) or a combination of QLD and abiraterone acetate for 24 h. After treatment, 10 μL of CCK-8 reagent (Abcam, Cat. No. ab228554) was added, followed by a 2-h incubation and measuring absorbance at 450 nm.

### QRT-PCR

Total RNA was extracted from PC3, DU145, PC3-AbiR, and DU145-AbiR cells using TRIzol reagent, and RNA concentration and purity were measured via a Nanodrop spectrophotometer. cDNA synthesis was performed using 1 μg of total RNA with the High-Capacity RNA-to-cDNA Kit. qRT-PCR was performed using SYBR Green PCR Master Mix on a real-time PCR system. miR-143 expression levels were normalized to U6 small nuclear RNA, and relative gene expression was analyzed via the 2^ΔΔ^*^Ct^* method.

### Western blot analysis

Cells were lysed in RIPA buffer containing protease and phosphatase inhibitors. Protein concentrations were measured using a BCA Protein Assay Kit, and equal amounts (30 μg) were resolved via 8% SDS-PAGE and transferred onto PVDF membranes.

Following blocking in 5% non-fat milk for 1 h, membranes were incubated overnight at 4 °C with primary antibodies against p-JNK (Abcam, Cat. No. ab307802; Dilution: 1:1000; Incubation condition: 4 °C, overnight), Bcl2 (Abcam, Cat. No. ab218123; Dilution: 1:1000; Incubation condition: 4 °C, overnight), p-Bcl2 (Abcam, Cat. No. ab32124; Dilution: 1:1000; Incubation condition: 4 °C, overnight), Beclin1 (Abcam, Cat. No. ab207612; Dilution: 1:2000; Incubation condition: 4 °C, overnight), and GAPDH (Abcam, Cat. No. ab8245; Dilution: 1:2000; Incubation condition: 4 °C, overnight). After washing, HRP-conjugated secondary antibodies were used to incubate the membrane for 1 h at room temperature. Protein bands were visualized using an ECL detection system, with densitometric analysis performed using ImageJ software.

### miR-143 mimic and inhibitor transfection

Abiraterone acetate-resistant PC3 and DU145-AbiR cells were transfected with miR-143 mimics (50 nM), miR-143 inhibitors (100 nM), or a corresponding negative control (NC) using Lipofectamine 2000, following the manufacturer’s protocol.

Cells seeded in six-well plates (3 × 105 cells/well) were incubated overnight. The following day, transfection complexes were prepared by mixing miRNA mimics or inhibitors with Lipofectamine 2000 in Opti-MEM and incubating for 20 min. The mixture was applied to cells for 24 or 48 h before analysis.

### Co-immunoprecipitation (Co-IP) assay

To investigate p-Bcl2-Beclin1 interactions, Co-IP assays were conducted. PC3-AbiR cells were lysed in ice-cold IP lysis buffer containing protease and phosphatase inhibitors. Lysates were pre-cleared with Protein A/G Sepharose beads for 1 h at 4 °C with gentle rotation.

For immunoprecipitation, lysates were incubated overnight at 4 °C with antibodies against p-Bcl2 or an IgG control. Protein A/G Sepharose beads were then added and incubated for 2 h at 4 °C. Immune complexes were washed three times, resuspended in SDS loading buffer, and analyzed via SDS-PAGE after anti-Beclin1 antibody incubation. ECL system was used to visualize bands, and ImageJ was used for densitometric analysis. Specificity was verified using IgG controls and input lysate controls.

### Statistical analysis

Results were reported as mean ± SD. GraphPad Prism 8 was used for statistical analysis. Differences between two groups were evaluated using a two-tailed Student’s *t*-test, while multiple group comparisons were analyzed using one-way ANOVA with Tukey’s *post-hoc* test. Significance was set at *p* < 0.05.

## Results

### Dysregulation of the miR-143/p-JNK/p-Bcl2-Beclin1 axis in abiraterone acetate-resistant prostate cancer cells

**FIGURE 1:**
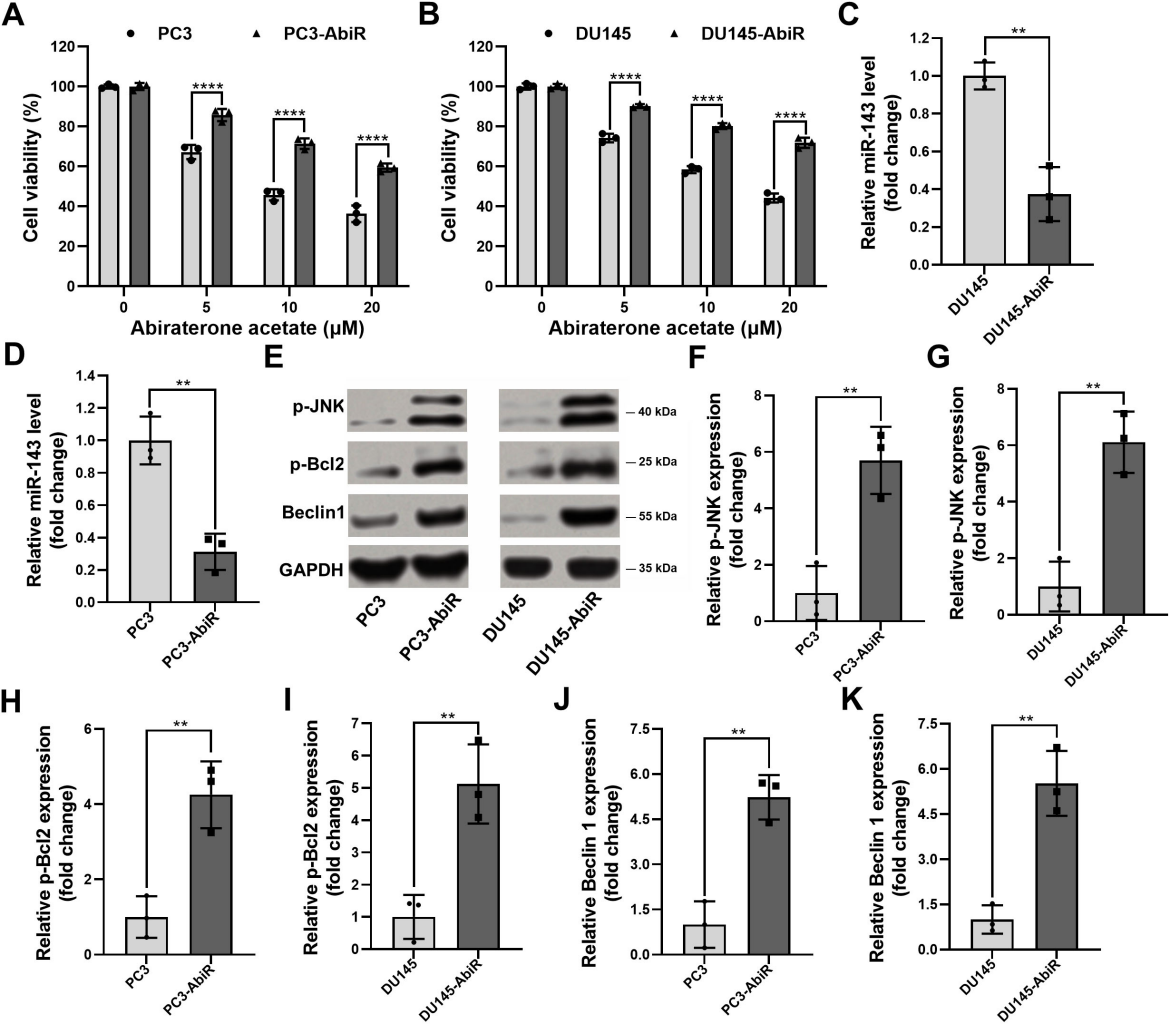
Dysregulation of miR-143/JNK/p-Bcl2-Beclin1 axis in abiraterone acetate-resistant prostate cancer cells. (A) PC3, abiraterone acetate-resistant PC3 (PC3-AbiR), (B) DU145 and abiraterone acetate-resistant DU145 (DU145-AbiR) cells were incubated with indicated increasing concentrations of abiraterone acetate for 24 h, followed by CCK-8 assay to assess the cell viability. (C) DU145, DU145-AbiR, and (D) PC3 and PC3-AbiR were prepared for qRT-PCR to assess the expression of miR-143. (E) PC3, PC3-AbiR, DU145 and DU145-AbiR cells were lysed for Western blot to assess the protein levels of p-JNK, p-Bcl2 and Beclin1, and (F–K) the optical density analyses were also measured. GAPDH was used as a loading control. Data were presented as the mean ± SD of three independent experiments. **p < 0.01, ****p < 0.0001.

To investigate the molecular mechanisms underlying abiraterone acetate resistance in prostate cancer, we established abiraterone acetate-resistant PC3 (PC3-AbiR) and DU145 (DU145-AbiR) cell lines. Cell viability assays using CCK-8 demonstrated that abiraterone acetate-resistant cells exhibited significantly higher survival rates than their parental counterparts upon abiraterone acetate treatment, indicating enhanced resistance (Figures 1A, B).

Given the role of microRNAs in drug resistance, we next analyzed the expression of miR-143, a known regulator of cancer progression. qRT-PCR analysis revealed a significant downregulation of miR-143 in PC3-AbiR and DU145-AbiR cells compared to their respective parental lines (Figures 1C, D). Since our previous studies indicated that miR-143 modulates the p-JNK/p-Bcl2-Beclin1 axis, we further assessed the activation status of this pathway. Western blot analysis showed increased phosphorylation of p-JNK and p-Bcl2, as well as elevated expression of Beclin1 in abiraterone acetate-resistant cells (Figures 1E–K).

### miR-143 modulates the p-JNK/p-Bcl2-Beclin1 axis in abiraterone acetate-resistant prostate cancer cells

**FIGURE 2:**
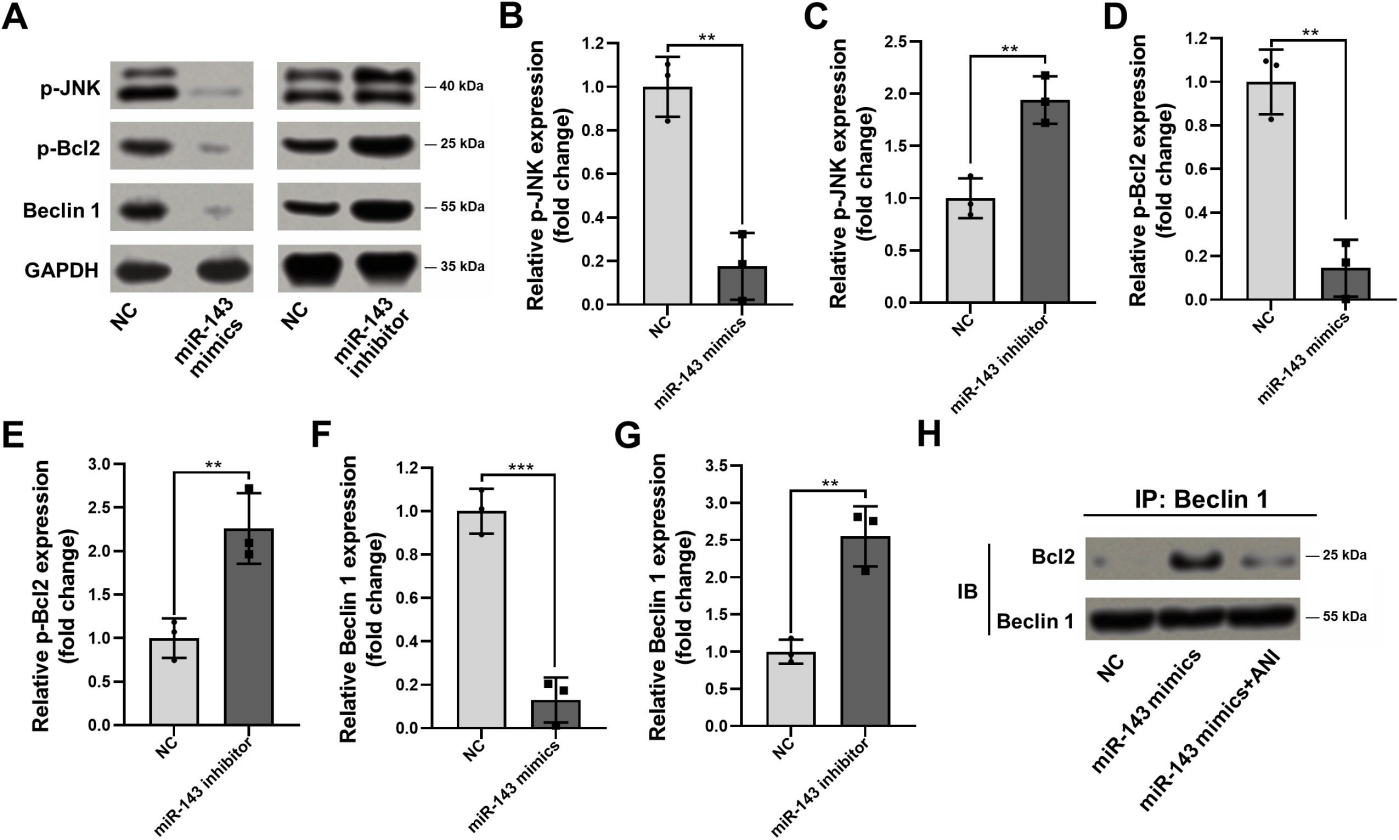
miR-143 modulates JNK/p-Bcl2-Beclin1 axis in abiraterone acetate-resistant prostate cancer cells. The miR-143 mimics, miR-143 inhibitor and corresponding control (NC) were respectively transfected into PC3-AbiR cells for 48 h. (A) The cells were then lysed for Western blot to assess the expression of p-JNK, p-Bcl2, Beclin1 and GAPDH, and (B–G) the optical density analyses were also measured. (H) PC3-AbiR cells transfected with NC or miR-143 mimics were incubated with vehicle or 5 ng/ml JNK agonist Anisomycin (ANI) for 24 h, followed by co-immunoprecipitation to assess the binding of p-Bcl2 and Beclin1 complex. IP, immunoprecipitation. IB, immunoblotting. Data were presented as the mean ± SD of three independent experiments. **p < 0.01, ***p < 0.001.

To determine whether miR-143 directly regulates the p-JNK/p-Bcl2-Beclin1 signaling axis, we modulated miR-143 levels in PC3-AbiR cells using miR-143 mimics or inhibitors. Western blot analysis showed that miR-143 overexpression significantly reduced the phosphorylation of p-JNK and p-Bcl2, along with a marked decrease in Beclin1 protein level. In contrast, inhibition of miR-143 led to the opposite effects, further supporting its role in regulating this pathway (Figures 2A–G).

Since p-Bcl2 is known to interact with Beclin1, we next examined whether miR-143 affects the formation of the p-Bcl2-Beclin1 complex. Co-immunoprecipitation assays demonstrated that miR-143 mimics enhanced p-Bcl2-Beclin1 binding, while activation of p-JNK attenuated this effect (Figure 2H). These results suggested that miR-143 plays a critical role in regulating the p-JNK/p-Bcl2-Beclin1 axis and may contribute to autophagy-associated mechanisms in abiraterone acetate-resistant prostate cancer cells.

### QLD enhances the effects of abiraterone acetate on miR-143 expression in abiraterone acetate-resistant cells

**FIGURE 3:**
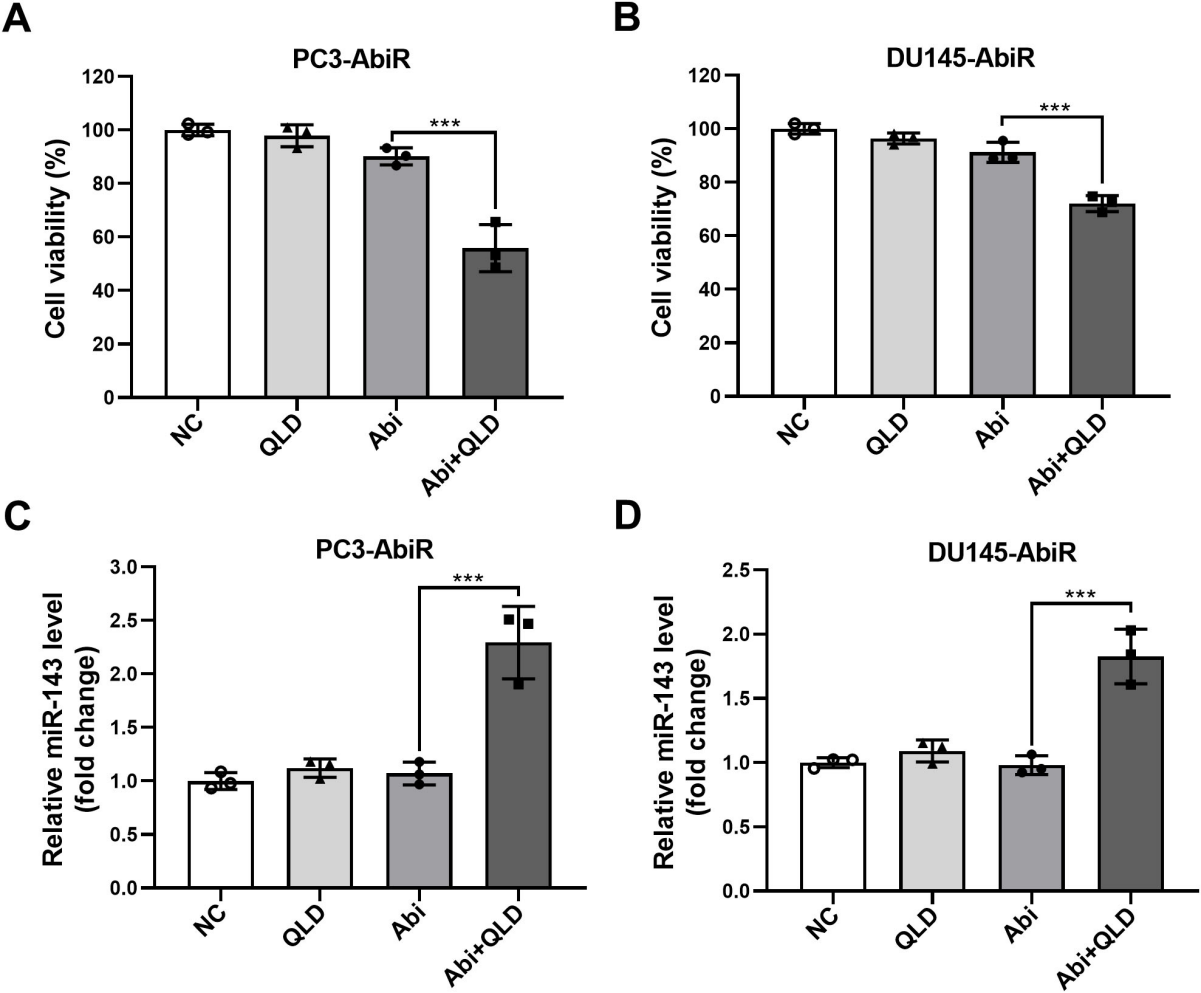
Qiling decoction (QLD) enhances the effects of abiraterone acetate on modulating miR-143 expression in abiraterone acetate-resistant prostate cancer cells. (A) PC3-AbiR and (B) DU145-AbiR cells were incubated with 10 mg/mL Qiling decoction (QLD) or 5 μM abiraterone acetate (Abi) for 24 h, followed by CCK-8 assay to assess cell viability. (C,D) Above cells were also prepared for qRT-PCR to assess the expression of miR-143. Data were presented as the mean ± SD of three independent experiments. ***p < 0.001.

Given the observed role of miR-143 in abiraterone acetate resistance, we sought to determine whether Qiling decoction (QLD), a traditional Chinese medicine formulation, could modulate miR-143 expression and sensitize resistant cells to abiraterone acetate treatment. CCK-8 assays revealed that QLD treatment significantly enhanced the cytotoxic effects of abiraterone acetate in PC3-AbiR and DU145-AbiR cells, suggesting a potential therapeutic benefit (Figures 3A, B, Supplementary Figure 1).

To further explore the mechanism of this enhanced sensitivity, we assessed miR-143 expression following QLD treatment. qRT-PCR analysis demonstrated that QLD significantly upregulated miR-143 levels in abiraterone acetate-resistant cell lines (Figures 3C, D). These findings suggest that QLD may exert its effects, at least in part, by restoring miR-143 expression, thereby reversing abiraterone acetate resistance.

### QLD modulates the p-JNK/p-Bcl2-Beclin1 axis in abiraterone acetate-resistant prostate cancer cells

**FIGURE 4:**
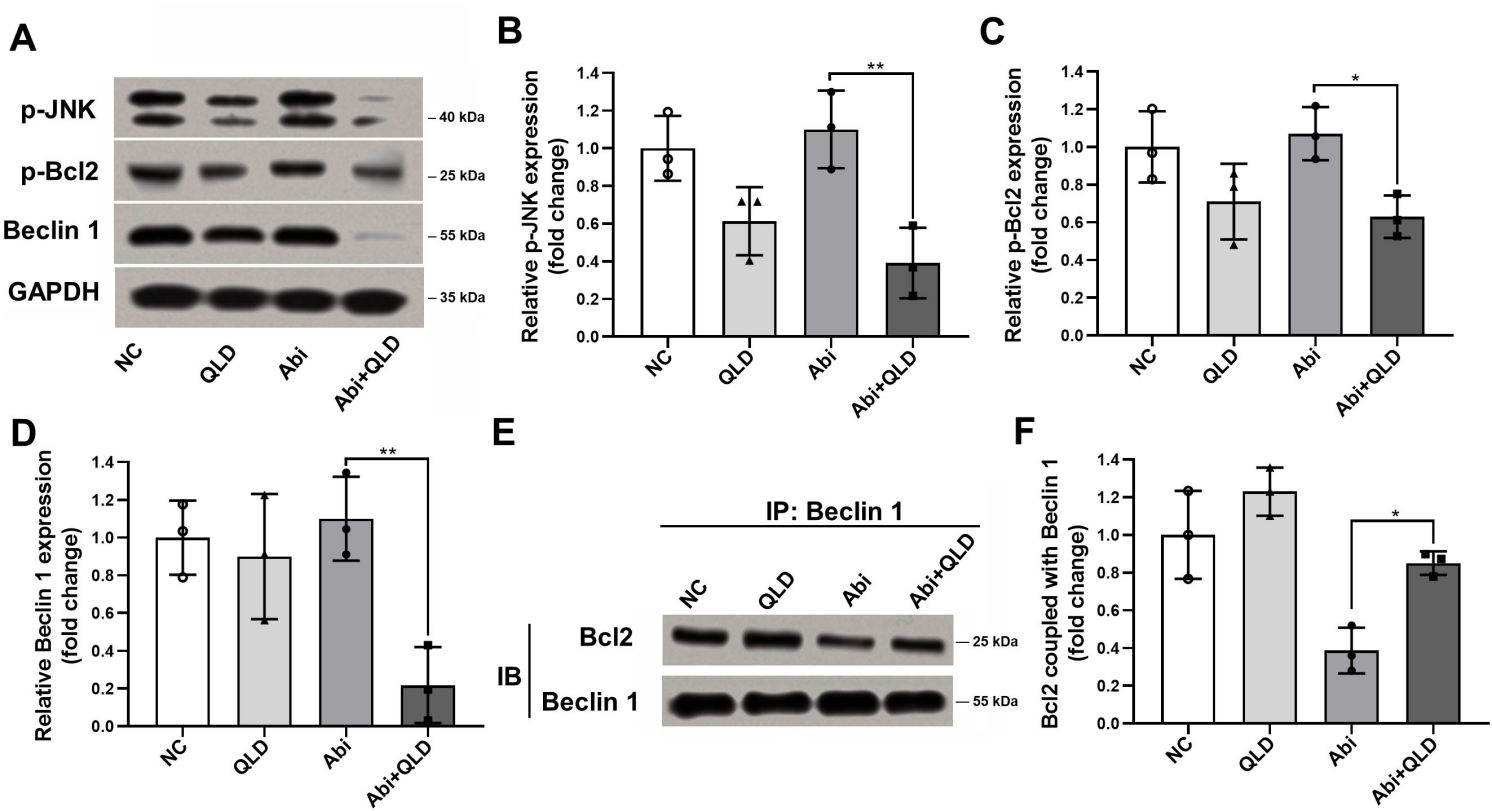
Qiling decoction (QLD) enhances the effects of abiraterone acetate on modulating JNK/p-Bcl2-Beclin1 axis in abiraterone acetate-resistant prostate cancer cells. PC3-AbiR cells were incubated with 10 mg/mL Qiling decoction (QLD) or 5 μM abiraterone acetate (Abi) for 24 h, followed by panel (A) Western blot to assess the protein levels of p-JNK, p-Bcl2 and Beclin1, and (B) the optical density analyses were also measured (B–D). GAPDH was used as a loading control. PC3-AbiR cells were incubated with 10 mg/mL QLD or 5 μM Abi for 24 h, followed by panel (E) co-immunoprecipitation to assess the binding of p-Bcl2 and Beclin1 complex, and (F) the optical density analyses were also measured. IP, immunoprecipitation. IB, immunoblotting. Data were presented as the mean ± SD of three independent experiments. *p < 0.05, **p < 0.01.

Since miR-143 negatively regulates the p-JNK/p-Bcl2-Beclin1 axis, we next investigated whether QLD affects this signaling pathway. Western blot analysis showed that QLD significantly suppressed the phosphorylation of p-JNK and p-Bcl2, while also decreasing Beclin1 protein levels in PC3-AbiR cells (Figures 4A–D). These findings indicate that QLD may function by inhibiting the p-JNK/p-Bcl2-Beclin1 axis, which is hyper-activated in abiraterone acetate-resistant cells.

Furthermore, we examined the formation of the p-Bcl2-Beclin1 complex to determine whether QLD enhances its assembly, a process that is known to suppress autophagy. Co-immunoprecipitation assays showed that QLD promoted p-Bcl2-Beclin1 complex formation in abiraterone acetate-resistant cells (Figures 4E, F). These results suggest that QLD not only restores miR-143 levels but also suppresses the p-JNK/p-Bcl2-Beclin1 axis, leading to reduced autophagy and increased sensitivity to abiraterone acetate.

### Inhibition of miR-143 abolishes the effects of QLD

**FIGURE 5:**
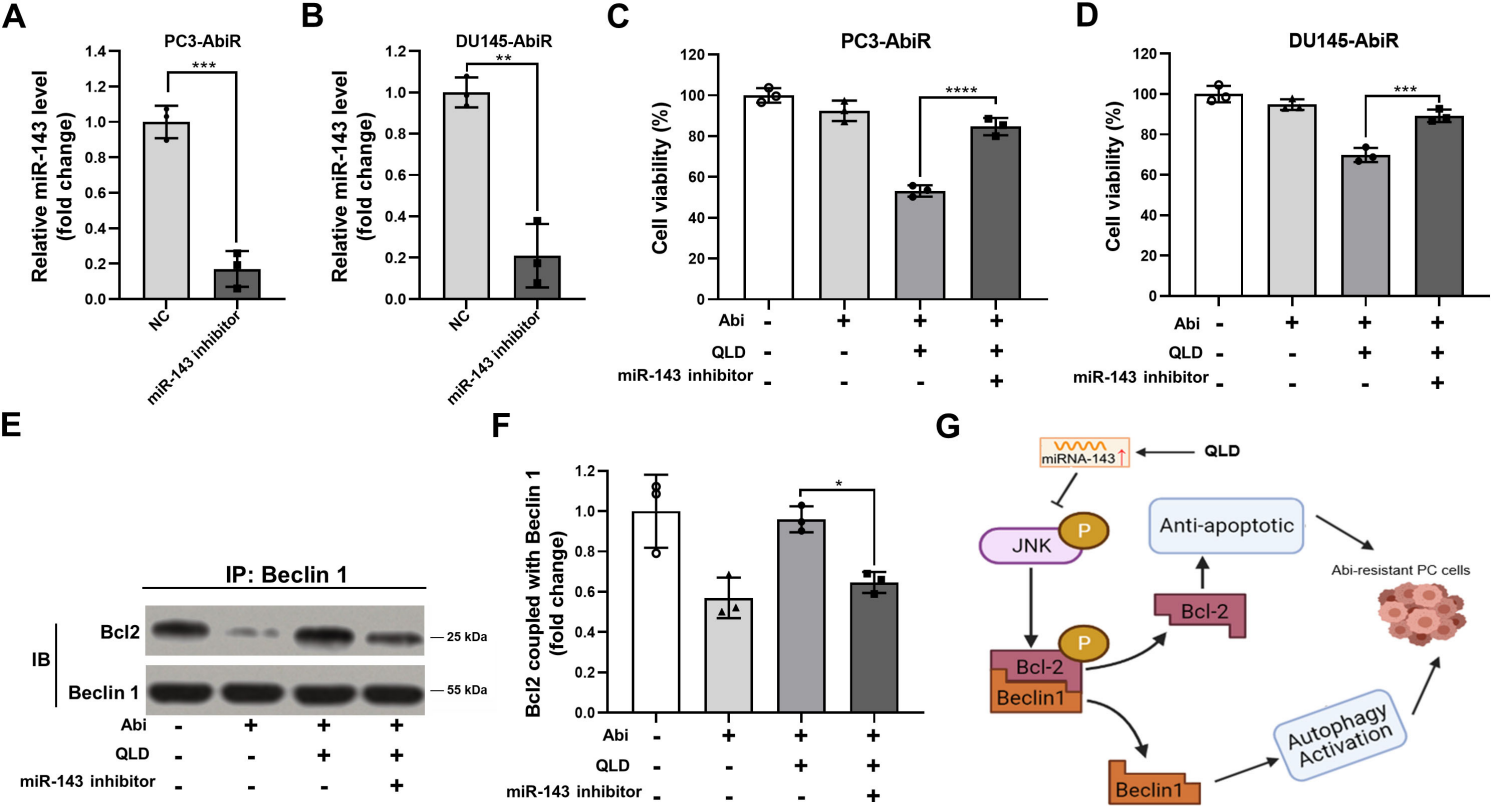
Inhibiting miR-143 abolishes the effects of QLD in abiraterone acetate-resistant prostate cancer cells. (A) PC3-AbiR and (B) DU145-AbiR cells were transfected with miR-143 inhibitor or normal control (NC) for 24 h, followed by qRT-PCR to assess the expression of miR-143. (C) PC3-AbiR and (D) DU145-AbiR cells transfected with NC or miR-143 inhibitor were incubated with 10 mg/mL QLD or 5 μM abiraterone acetate (Abi) for 24 h, followed by CCK-8 assay. PC3-AbiR cells transfected with NC or miR-143 inhibitor were incubated with 10 mg/mL QLD or 5 μM Abi for 24 h, followed by panel (E) co-immunoprecipitation to assess the binding of p-Bcl2 and Beclin1 complex, and (F) the optical density analyses were also measured. (G) The mechanism diagram illustrating how QLD induces prostate cancer (PC) cells to overcome abiraterone resistance. Data were presented as the mean ± SD of three independent experiments. *p < 0.05, **p < 0.01, ***p < 0.001, ****p < 0.0001.

To confirm that QLD exerts its effects through miR-143, we knocked down miR-143 in PC3-AbiR and DU145-AbiR cells using miR-143 inhibitors before treating the cells with QLD and abiraterone acetate. qRT-PCR analysis confirmed that miR-143 inhibitors effectively suppressed miR-143 expression (Figures 5A, B).

Cell Counting Kit-8 assays showed that inhibition of miR-143 significantly diminished the cytotoxic effects of QLD and abiraterone acetate co-treatment, indicating that miR-143 is required for QLD-mediated sensitization to abiraterone acetate (Figures 5C, D). Additionally, co-immunoprecipitation assays revealed that miR-143 inhibition disrupted QLD-induced p-Bcl2-Beclin1 complex formation, further supporting the role of miR-143 in regulating autophagy through this pathway (Figures 5E, F). In addition, the mechanism diagram illustrating how QLD induced prostate cancer cells to overcome abiraterone resistance was shown as in Figure 5G.

## Discussion

In this study, we investigated the role of miR-143 in abiraterone acetate-resistant prostate cancer and its regulatory effects on the p-JNK/p-Bcl2-Beclin1 signaling axis. Our results demonstrated that miR-143 is significantly downregulated in abiraterone acetate-resistant PC3-AbiR and DU145-AbiR cells, suggesting its potential involvement in drug resistance. We further found that JNK and p-Bcl2 phosphorylation, along with Beclin1 expression, were elevated in resistant cells, indicating activation of the p-JNK/p-Bcl2-Beclin1 axis. These findings suggest that the loss of miR-143 contributes to abiraterone acetate resistance by promoting autophagy-mediated survival signaling, echoing previous findings (23, 24). Importantly, treatment with QLD restored miR-143 expression, inhibited the p-JNK/p-Bcl2-Beclin1 axis, and enhanced the sensitivity of resistant cells to abiraterone acetate, providing a potential therapeutic strategy for overcoming resistance (25).

Our findings align with previous studies highlighting the tumor-suppressing role of miR-143 (26). Several reports have indicated that miR-143 expression is reduced in castration-resistant prostate cancer and is associated with enhanced tumor growth, increased metastatic potential, and resistance to chemotherapy. Previous studies have shown that miR-143 targets oncogenic pathways such as ERK/MAPK, PI3K/AKT, and androgen receptor signaling, all of which contribute to prostate cancer progression and drug resistance (27, 28). However, few have focused on the relationship between miR-143 and autophagy in abiraterone acetate resistance. This study fills that gap by identifying the p-JNK/p-Bcl2-Beclin1 axis as a downstream target of miR-143 and demonstrating that dysregulation of this pathway promotes autophagy-mediated resistance.

In prostate cancer, autophagy has been implicated in resistance to androgen receptor-targeting therapies, including abiraterone and enzalutamide. Our findings support previous findings that inhibition of miR-143 led to increased activation of the p-JNK/p-Bcl2-Beclin1 axis and enhanced autophagy, thereby promoting cell survival in the presence of abiraterone acetate (23). Conversely, restoration of miR-143 suppressed this pathway and sensitized resistant cells to abiraterone acetate, suggesting that targeting autophagy through miR-143 regulation may be a viable therapeutic approach. This study highlights a novel mechanism of resistance and suggests that miR-143 restoration, either through direct miRNA replacement or pharmacological modulation using QLD, could serve as a promising strategy to enhance abiraterone efficacy.

Qiling decoction has been shown in clinical practice to exhibit a strong safety profile, with minimal toxicity and no significant adverse events reported in patients with cancer or other chronic conditions (20). Previous studies have demonstrated that QLD does not induce hepatic or renal dysfunction, nor does it exacerbate hematologic parameters, making it suitable for long-term use (20). This favorable safety margin supports its potential integration alongside abiraterone in prostate cancer treatment, especially in cases requiring extended therapeutic duration. Potential drug–herb interactions between QLD and abiraterone acetate warrant consideration. Although no adverse effects have been reported, the complex herbal composition of QLD could theoretically influence abiraterone metabolism via cytochrome P450 modulation. Future pharmacokinetic and clinical studies are needed to confirm the safety of this combination therapy. QLD has been traditionally used in Chinese medicine for its anti-cancer and immune-regulatory properties. However, its role in reversing abiraterone acetate resistance in prostate cancer has not been previously explored. Our findings indicate that QLD exerts its effects by restoring miR-143 expression, thereby inhibiting the p-JNK/p-Bcl2-Beclin1 axis and reducing autophagy-mediated resistance, as a novel mechanism of action. These results provide a strong rationale for further preclinical and clinical studies to evaluate its therapeutic potential as an adjunct to abiraterone acetate treatment.

Beyond its molecular mechanisms, the findings of this study also align with the broader framework of integrating traditional Chinese medicine (TCM) with modern biomedicine. A recent review highlights the growing emphasis on evidence-based validation of traditional formulations through molecular and clinical research (29). By demonstrating how QLD modulates miRNA signaling and enhances abiraterone efficacy, our work provides a concrete example of this integrative approach. This not only supports the scientization of TCM but also underscores the translational potential of QLD as a bridge between traditional theory and modern oncology practice.

Several limitations should be acknowledged for our study. First, the experiments were conducted *in vitro* using cell line models, which may not fully recapitulate the complexity of resistance mechanisms in patients. Future studies should include *in vivo* models to validate the therapeutic effects of miR-143 restoration and QLD treatment. Second, although the p-JNK/p-Bcl2-Beclin1 axis was identified as a key pathway regulated by miR-143, other potential targets of miR-143 in abiraterone acetate resistance remain to be explored. Comprehensive transcriptomic and proteomic analyses could help identify additional downstream effectors. Finally, the precise components of QLD responsible for its effects on miR-143 expression and autophagy inhibition require further investigation. Identifying the active compounds within this formulation could facilitate the development of more targeted therapeutic strategies.

In future work, emerging methodologies such as artificial intelligence (AI)-assisted omics integration could offer new opportunities to deepen mechanistic understanding and optimize traditional formulations. As discussed in a recent paper (30), AI-driven transcriptomic and metabolomic analyses may help identify novel miR-143 targets and clarify the synergistic interactions among QLD components. Incorporating these computational approaches could enhance the evidence-based modernization of QLD and accelerate its translational development within the framework of precision oncology.

## Conclusion

In summary, this study demonstrates that downregulation of miR-143 contributes to abiraterone acetate resistance in prostate cancer by activating the p-JNK/p-Bcl2-Beclin1 signaling axis and promoting autophagy. Restoring miR-143 expression, either through miRNA-based therapy or pharmacological intervention with QLD, enhances the sensitivity of resistant cells to abiraterone acetate. These findings provide new insights into the molecular basis of abiraterone acetate resistance and suggest a novel therapeutic strategy for improving treatment outcomes in prostate cancer. Further studies, including *in vivo* validation and clinical trials, are warranted to translate these findings into potential clinical applications.

## Data Availability

The datasets presented in this study can be found in online repositories. The names of the repository/repositories and accession number(s) can be found in the article/Supplementary material.
