# Supplementary material for: Qiling decoction enhances the anti-tumor activity of abiraterone acetate by up-regulating miR-143 expression in abiraterone acetate-resistant prostate cancer cells
Source: Front Med (Lausanne). 2025 Dec 9;12:1643506. doi: 10.3389/fmed.2025.1643506 (PMC12722432; doi:10.3389/fmed.2025.1643506)
Supplement: Supplementary file 1 [file Image_1.pdf]

Supplementary materials

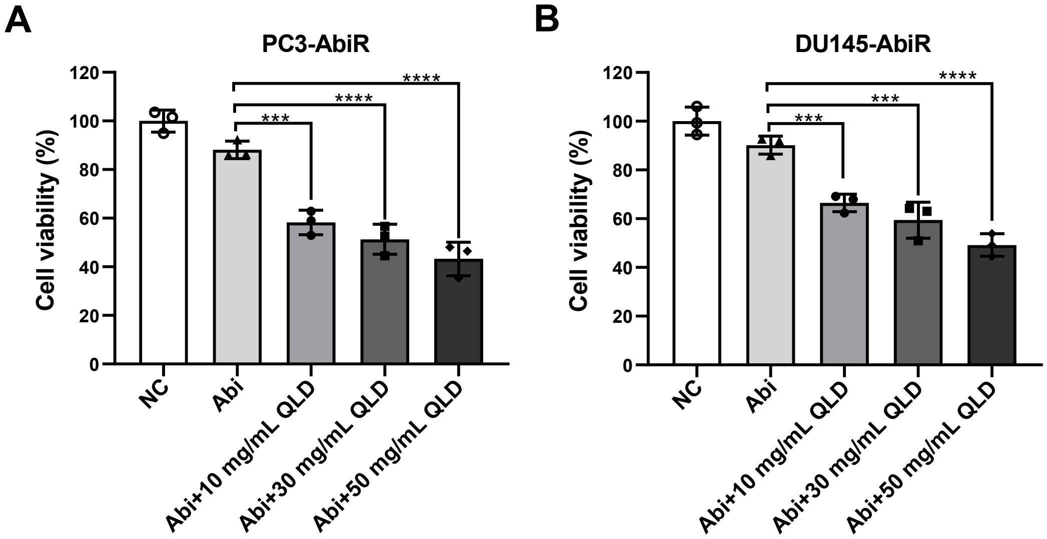

Figure S1. QLD enhances the effects of abiraterone acetate in abiraterone acetate-resistant prostate cancer cells. PC3-AbiR (A) and DU145-AbiR (B) cells were incubated with indicated Qiling decoction (QLD) or 5 μM abiraterone acetate (Abi) for 24 hours, followed by CCK-8 assay to assess cell viability. $^{***}p < 0.001$, $^{****}p < 0.0001$.
